# Communities at the extreme: Aquatic food webs in desert landscapes

**DOI:** 10.1002/ece3.5648

**Published:** 2019-09-12

**Authors:** Nicholas P. Moran, Bob B. M. Wong, Ross M. Thompson

**Affiliations:** ^1^ School of Biological Sciences Monash University Clayton Vic. Australia; ^2^ Evolutionary Biology Bielefeld University Bielefeld Germany; ^3^ Institute for Applied Ecology University of Canberra Canberra ACT Australia

**Keywords:** community, food webs, ground water, stable isotopes, temporary pools

## Abstract

Studying food webs across contrasting abiotic conditions is an important tool in understanding how environmental variability impacts community structure and ecosystem dynamics. The study of extreme environments provides insight into community‐wide level responses to environmental pressures with relevance to the future management of aquatic ecosystems. In the western Lake Eyre Basin of arid Australia, there are two characteristic and contrasting aquatic habitats: springs and rivers. Permanent isolated Great Artesian Basin springs represent hydrologically persistent environments in an arid desert landscape. In contrast, hydrologically variable river waterholes are ephemeral in space and time. We comprehensively sampled aquatic assemblages in contrasting ecosystem types to assess patterns in community composition and to quantify food web attributes with stable isotopes. Springs and rivers were found to have markedly different invertebrate communities, with rivers dominated by more dispersive species and springs associated with species that show high local endemism. Qualitative assessment of basal resources shows autochthonous carbon appears to be a key basal resource in both types of habitat, although the particular sources differed between habitats. Food‐web variables such as trophic length, trophic breadth, and community isotopic niche size were relatively similar in the two habitat types. The basis for the similarity in food‐web structure despite differences in community composition appears to be broader isotopic niches for predatory invertebrates and fish in springs as compared with rivers. In contrast to published theory, our findings suggest that the food webs of the hydrologically variable river sites may show less dietary generalization and more compact food‐web modules than in springs.

## Graphical abstract

We analyzed aquatic assemblages in the western Lake Eyre Basin of arid Australia, using stable isotope analysis and measures of community composition across two characteristic and contrasting aquatic habitats: springs and rivers. Springs and rivers were found to have markedly different invertebrate communities, but general food‐web variables such as trophic length, trophic breadth, and community isotopic niche size were relatively similar in the two habitat types despite some subtle differences in the isotopic niche of specific functional groups and basal carbon sources.

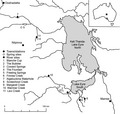

## 1 INTRODUCTION

Studying ecological communities across environmental gradients is a key step toward predicting how complex communities respond to their abiotic environment, providing insight into how they persist over time (Dunne, Saleska, Fischer, & Harte, 2004; Thompson, Dunne, & Woodward, 2012). Changes along environmental gradients can alter which taxa are present and their abundance (community composition) and the trophic interactions between taxa (food‐web structure). Food‐web analysis includes all species in the system and the movement of energy between those species (Cole et al., 2006; Thompson, Brose, et al., 2012). By including all species present and their interactions, we can understand how whole ecosystems respond to stressors such as invasive species (Ho, Bond, & Lake, 2011), drought (Ledger, Brown, Edwards, Milner, & Woodward, 2013), or climate change (Binzer, Guill, Brose, & Rall, 2012). Instead of focusing on effects on individual species, food‐web approaches can account for emergent and interactive effects of stressors on whole communities (Layer et al., 2011).

Analysis of carbon and nitrogen isotopes quantifies the relative trophic positions of species, which allows the structure of the food web to be characterized and emergent food‐web variables to be quantified (Post, 2002). For example, food‐chain length (i.e., the number of trophic transfers from basal resources to top predators) is a commonly studied response metric that can assess the factors that support high trophic‐level species, overall community complexity, and biodiversity (McHugh, McIntosh, & Jellyman, 2010). Similarly, the ecological niche occupied by functional groups and species can be quantified using the “isotopic niche” concept, where the isotope composition of an organism's tissue reflects what it consumes (Jackson, Inger, Parnell, & Bearhop, 2011; Newsome, Martinez del Rio, Bearhop, & Phillips, 2007). The size and shape of the niche occupied by taxa or functional groups can be a sensitive measure of community trophic characteristics under varying levels of abiotic stress (McGill, Enquist, Weiher, & Westoby, 2006).

In aquatic ecosystems, hydrology is a key ecological driver which can influence community composition both directly through disturbance effects (Bunn & Arthington, 2002; Lake, 2003) and indirectly through impacts on basal resource availability (Takimoto & Post, 2013; Townsend et al., 1998). Natural disturbance, such as the extreme high flow events and droughts experienced by desert rivers, has been hypothesized as a key driver of patterns of diversity generally, although empirical evidence remains weak for classical ecological hypotheses such as the “intermediate disturbance hypothesis” (Connell, 1978; Fox, 2013). More disturbed habitats may be more likely to contain species with broad diets, including omnivores (e.g., Menge & Sutherland, 1987). This hypothesis is supported by empirical studies of early successional communities, where trophic generalists are more common (e.g., Brown & Southwood, 1983; Kullberg & Scheibe, 1989). Applying stable isotope analysis to communities' subject to differences in flow permanence allows us to assess how disturbance may influence total niche width and isotopic niche size (Jackson et al., 2011).

The effects of disturbance on food‐web characteristics have long been of interest to ecologists, particularly from a perspective of understanding food‐web attributes that may promote resilience and resistance (e.g., Kondoh, 2003; Krause, Frank, Mason, Ulanowicz, & Taylor, 2003; Wootton, Parker, & Power, 1996). It has been hypothesized that more variable habitats will have shorter food chains, due to stochastic loss of numerically rare top predators (McHugh et al., 2010; Pimm & Lawton, 1977). Experimental support for this found negative effects of hydrologic variability on food‐chain length in North American rivers (Sabo, Finlay, Kennedy, & Post, 2010). However, Walters and Post (2008) found that a low‐flow disturbance altered the body size characteristics of communities but not the length of the food chain. A recent meta‐analysis also found little evidence of an effect of disturbance on food‐chain length (Takimoto & Post, 2013).

Indirect effects of disturbance on diversity through impacts on the basal resource supply have received relatively less attention (Wootton, 1994a, 1994b; but see Townsend et al., 1998). Across aquatic ecosystems, and particularly in river systems, basal carbon from in‐stream autochthonous (e.g., algal and macrophyte) and allochthonous (e.g., riparian and floodplain) sources are fundamental processes underpinning food webs (Bunn, Davies, & Winning, 2003; Cole et al., 2006). Carbon isotope analysis allows for tracking basal resources such as algal or terrestrial carbon through food webs (Post, 2002). Isotope studies of primary productivity have provided unique insights into the relative influence of carbon sources, where flow variability can play an important role in facilitating inputs of terrestrial riparian and floodplain carbon (Bunn et al., 2003; Hadwen, Fellows, et al., 2010; Robertson, Bunn, Boon, & Walker, 1999).

Two aquatic ecosystems in arid Australia provide an opportunity to analyze whether differences in flow permanence and abiotic variability influence community composition and food‐web structure. In northern South Australia, the Great Artesian Basin (GAB) forms a series of highly stable and extremely isolated groundwater springs (Mudd, 2000; Murphy, Breed, Guzik, Cooper, & Austin, 2012), while the Lake Eyre Basin (LEB) is a largely ephemeral surface water basin that includes some of the most hydrologically variable rivers in the world (Kotwicki & Allan, 1998; McNeil, Schmarr, & Rosenberger, 2011). Western LEB waterholes range from semi‐permanent to highly ephemeral, and the flow variation is coupled with variation in physiochemical properties; for example, salinity can range from near fresh during high flows to hypersaline in recession phases (Costelloe, Grayson, McMahon, & Argent, 2005; Costelloe & Russell, 2014). Biodiversity studies have shown that assemblages have been highly conserved in arid Australian waterholes over time, with more persistent waterholes being key ecological refugia (Davis, Pavlova, Thompson, & Sunnucks, 2013). Furthermore, studies have shown that some taxa are reliant on GAB waterbodies and show high spring‐ and catchment‐specific endemism (Murphy, Adams, & Austin, 2009; Murphy et al., 2012), and permanent springs across arid Australia broadly tend to be characterized by endemic species in contrast to the riverine communities (Davis et al., 2018). This suggests that present‐day assemblages are likely to be the product of long‐term physical differences between waterbodies and their connectivity within the landscape, and the contrasting abiotic regimes provide an opportunity to study their influence on community characteristics.

We integrated community composition analysis with stable isotope approaches (Layman et al., 2012) and contemporary methods to quantify isotopic niche size (Jackson et al., 2011). This approach was used to explore how physical variability influences aquatic food‐web characteristics (Thompson, Brose, et al., 2012). Specifically, we sought to determine whether the differences in the abiotic characteristics of river and spring habitats influence:

1. Community composition, where we predicted that river environments would be characterized by broadly dispersed, highly mobile species, whereas spring habitats would contain taxa with poorer dispersal abilities and have higher local endemism.
2. Basal resource base, where we predicted that river environments may have a broader resource base due to lateral connectivity with riparian and floodplain energy sources.
3. Isotopic niche size of the community, which we predicted would be larger in more disturbed sites due to selection for generalist taxa.
4. Food‐chain length, where we predicted that food chains would be shorter in the more physical‐disturbance prone river food webs compared to the more stable spring food webs.

## 2 METHODS

### 2.1 Study location

**Figure 1:**
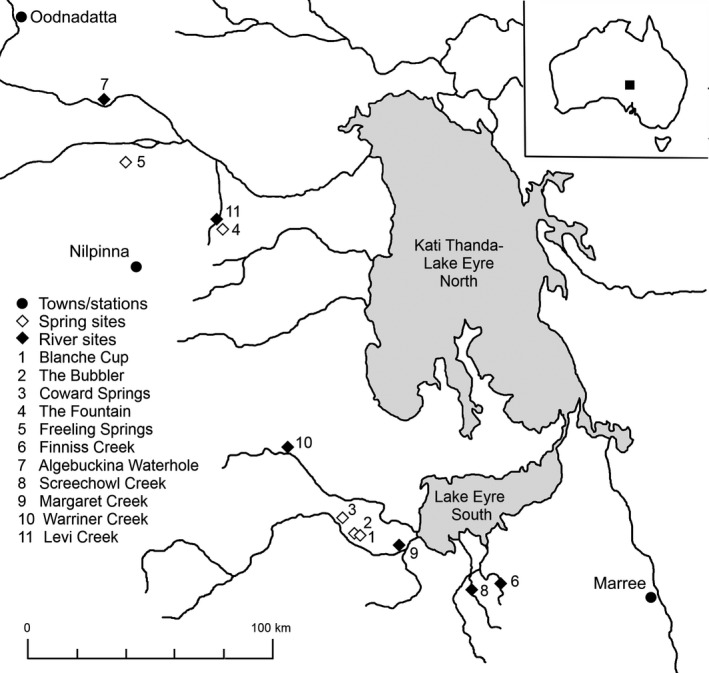
Map of the 11 study sites in northern South Australia, within the western Lake Eyre Basin. Both spring (sites 1–5) and river (sites 6–11) sites span multiple river systems that feed into Lake Eyre, with the northern sites (4, 5, 7, 11) within the Neales River Catchment, and the remaining sites in smaller creek catchments that flow into the lake's southern lagoon. 2013–2014 rainfall records at towns/stations shown are available in Appendix S1 (Figures A1–A3)

Sites were selected to represent permanent groundwater springs (*n* = 5) and intermittent river waterholes spanning multiple catchments in the western Lake Eyre Basin (*n* = 6; Table 1, Figure 1). Spring sites chosen were the largest accessible springs in the area, while waterholes chosen were the largest known accessible pools to maximize the chance that sites were not dry during sampling. Both aquatic habitat types span a range of sizes and to ensure consistent areas were sampled, entire pools/spring outlets were sampled where they were <50 m length and a maximum 50 m reach was sampled for larger sites (e.g., Algebuckina, The Bubbler). In April 2014, invertebrate and fish communities were sampled as well as basal resources, including plant material from riparian and aquatic vegetation, phytoplankton, and algae. At sampling, all river sites were nonflowing pools, with physiochemical properties showing high among‐ and within‐site variation relative to springs during the study period and measured parameters (e.g., temperature, conductivity, salinity) ranging from below that of spring sites to significantly higher (Table 1). A rainfall event in February 2014 impacting the entire region (although potentially more strongly in southern sites; see Appendix S1 for 2013–2014 rainfall data, Figures A1–A3) may have temporarily provided increased longitudinal connectivity in river sites, despite most ephemeral sites (i.e., Screechowl, Margaret, and Levi Creeks) likely being almost entirely dry prior to that rainfall.

**Table 1:** Ecological characteristics of study sites. Water quality data collected in autumn (March–May) annually from 2013 to 2015

Site name	Latitude/longitude	Habitat	Conductivity mean/range (μS/cm^2^)	Temperature mean/range (°C)	Turbidity mean/range (NTU)
Blanche Cup	−29.452850/136.858733°	Spring	7,100 (6,809–7,270)	20.9 (20.2–21.7)	17.3 (10.9–25.9)
The Bubbler	−29.446483°/136.857849°	Spring	5,746 (5,490–6,127)	29.7 (29.1–30.5)	7.22 (7.11–7.36)
Coward Springs	−29.400388°/136.794193°	Spring	6,769 (6,550–6,980)	24.3 (22.2–26.9)	6.44 (4.72–9.00)
The Fountain	−28.348694°/136.283585°	Spring	6,318 (6,233–6,390)	21.4 (18.5–23.7)	9.54 (8.09–11.9)
Freeling Springs	−28.071861°/135.903622°	Spring	8,433 (8,180–8,598)	22.3 (19.6–24.5)	4.76 (1.98–3.70)
Finniss Creek	−29.610250°/137.458289°	River	12,900 (12,690–13,030)	23.0 (18.6–26.9)	6.23 (3.50–8.06)
Algebuckina	−27.899995°/135.814456°	River	8,200 (2,900–12,150)	23.9 (12.9–32.3)	35.1 (8.68–59.8)
Screechowl Creek	−29.627539°/137.336036°	River	143,800 (73,700–231,000)	21.9 (15.4–28.3)	94.8 (19.3–224)
Margaret Creek	−29.490222°/137.039383°	River	118,330 (26,100–227,300)	23.1 (17.7–29.5)	76.3 (9.97–172)
Warriner Creek	−29.137986°/136.568422°	River	57,268 (35,400–78,704)	20.7 (19.2–22.1)	6.31 (4.28–9.04)
Levi Creek	−28.317600°/136.270933°	River	182,980 (100,600–265,360)	25.7 (17.6–33.7)	14.3 (11.9–16.6)

### 2.2 Stable isotope sampling and processing

For basal resources, three replicate samples (predominantly leaves) were collected from all plant species observed at sites and frozen. After transport to Monash University, samples were rinsed with reverse osmosis (RO) water and oven‐dried for 1 week at 60°C. Samples were then ground to a fine powder using a mortar and pestle (HCl 1 N acid‐washed) or a mill grinder. Phytoplankton/FPOM samples (100 ml) were collected using a 40‐µm mesh plankton sampling net (25 cm opening, 5 m sweeps). Samples were frozen for storage and transport before being prefiltered through a 125‐µm sieve and vacuum‐filtered onto a GF‐75 filter (0.7 µm mesh size; Advantec MFS Inc.). Filters were rinsed with 1 N HCl to remove carbonates and rinsed well with RO water, before drying for 1 week at 60°C. Algal resources, particularly filamentous or benthic mats, could be sampled by hand. Replicate samples were rinsed with RO water, and all invertebrates and other organic matter were removed under a dissecting microscope before the algae was dried and ground.

Invertebrate composite samples were collected by scooping a dip net through benthic sediment and the water column (two sweep and one kick sample per site), excluding Blanche Cup where three sweeps were collected as the delicate banks did not allow kick samples. Composite samples were rinsed with RO water and held for 24 hr before freezing to allow invertebrate gut contents to be voided, to avoid biasing isotope values with undigested gut material that may not ultimately be assimilated into tissue. Invertebrates were identified to the finest possible taxonomic level, generally family or species, and their presence/absence in each sample was recorded. Gastropods, which were common, had their shells removed before processing, as carbon‐based precipitates reflect the isotopic ratios of the inorganic environment, whereas their soft tissue reflects their diet (Post, 2002). Where enough biomass was available, three independent (distinct individuals/groups of individuals) replicates were analyzed. If insufficient biomass was sampled, samples were pooled to give a mean value per species/site. Replicates were dried and ground before analysis. For fish and crayfish sampling, differences in the depth between sites did not allow a standardized approach to be used. In springs, unbaited box traps (45 cm × 25 cm × 25 cm, 5 cm aperture, 1 mm mesh) and hand dip nets (20 cm × 25 cm, 1 mm mesh) were used. At river sites, three fkye nets (single 5 m wing, 60 cm drop, 19 mm mesh, deployed overnight) and a seine net (7 m × 2 m, 6 mm mesh) were also used, water depth permitting. Individuals were removed and euthanized by immersion in a solution of clove oil and water sourced from the site, before freezing. Where enough individuals were collected, three independent samples of muscle tissue were rinsed, dried, and ground before analysis. If there were insufficient individuals/biomass to provide three independent samples, three samples were analyzed where possible and a mean isotope value reported.

### 2.3 Stable isotope analysis

Samples were analyzed for carbon (carbon‐12–carbon‐13; hereafter δ^13^C) and nitrogen (nitrogen‐14–nitrogen‐15; hereafter δ^15^N) isotope ratios at the Water Studies Centre (Monash University) on an ANCA GSL2 elemental analyzer interfaced to a Hydra 20–22 continuous‐flow isotope ratio mass‐spectrometer (Sercon Ltd.). The precision of the stable isotope analysis was ±0.1‰ for δ^13^C and ±0.2‰ for δ^15^N. Stable isotope data are expressed in the delta notation (δ^13^C and δ^15^N), relative to the stable isotopic ratio of Vienna Pee Dee Belemnite standard (*R*_VPDB_ = 0.0111797) for carbon and atmospheric N_2_ (*R*_Air_ = 0.0036765) for nitrogen.

### 2.4 Statistical analysis

All statistical analyses were conducted using R Statistical Package 3.5.1 (R Core Team, 2018). To analyze community composition, we used invertebrate taxa presence/absence data by site in multidimensional scaling (MDS) analysis. Invertebrate data (cf. fish) were used as we were able to apply a more strictly standardized sampling methodology across sites and because the desert goby (*Chlamydogobius eremius*) is known to be the only species present in any spring site. Only taxa caught using kick/sweep sampling were used in this analysis, while all taxa (including those captured using other methods, e.g., *Cherax destructor*) are used in isotope analysis. PERANOVA hypothesis tests (adonis function, vegan package; Oksanen et al., 2007) tested for differences in composition associated with habitat type. We also tested for an effect of region as genetic isolation between northern (e.g., Algebuckina) and southern (e.g., Warriner Creek) catchments has been detected in previous studies (see Mossop et al., 2015). As compositional differences were found between springs and rivers, the contribution of each taxa to the effect of habitat type was estimated using Bray–Cutis dissimilarity (SIMPER function). We considered taxa with a percentage contribution greater than its standard deviation to be substantially contributing to the habitat differences in composition, and their taxa‐specific NMDS scores were calculated.

In addition to the descriptive analysis of stable isotope plots, the sample size‐corrected standard ellipse area (SEAc; Jackson et al., 2011) was calculated at each site for all animal taxa collectively as well as separately by functional feeding groups. Coarse functional groupings for aquatic invertebrate were made at the finest taxonomic level available by (a) nonpredatory invertebrates (including scrapers, gathering and filtering collectors, and shredders) and (b) predatory invertebrates and fish (via the MDFRC Bug Guide; Hawking, Smith, LeBusque, & Davey, 2013). Predatory invertebrates and fish were grouped out of necessity as most sites lacked sufficient taxa to calculate SEAc for each group separately, and this grouping gives a measure of the trophic niche occupied by species that we are assuming to be feeding at higher (>2) trophic levels. SEAc represents size of the 40% prediction interval of the posterior distribution derived from an input set of isotope values, to give an estimate of isotopic niche size, whereas convex hull area measures the area of a polygon encompassing all input isotope values (Jackson et al., 2011). Additional food‐web attributes were calculated for animal communities, such as the δ^15^N range (community trophic range taken as the difference from the maximum to mean basal δ^15^N, a nontransferable but similar measure to food‐chain length), δ^13^C range (community trophic breadth), mean δ^15^N (corrected for basal δ^15^N, to estimate mean trophic position of taxa, Vander Zanden, Cabana, & Rasmussen, 1997), mean δ^13^C, and total number of taxa. One‐way ANOVAs compared each measure between spring and river sites.

## 3 RESULTS

**Figure 2:**
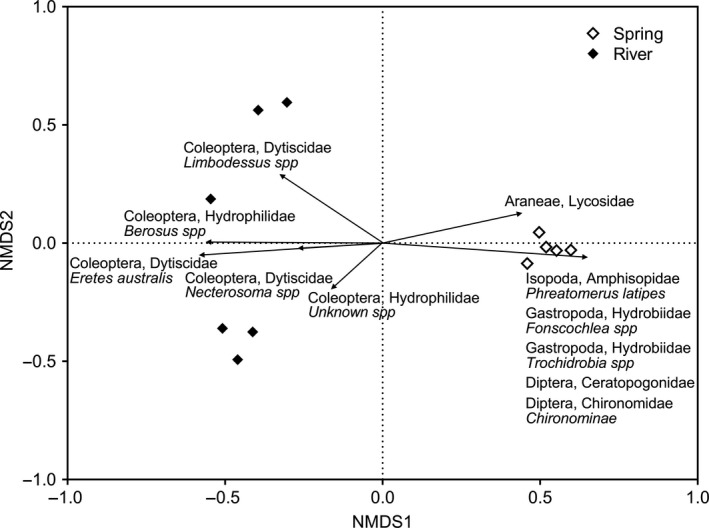
Invertebrate community March–April 2014 presence/absence multidimensional scaling plot. Symbols represent sites, and vectors represent individual invertebrate groupings that significantly contributed to the habitat divergence. Vectors show the NMDS scores for those taxa

We found a significant effect of habitat type on invertebrate community composition (*F*_1,8_ = 12.1370, *p* = .001, Figure 2) and no effect of region. Eleven invertebrate taxa were found to be substantially contributing to the habitat effect via SIMPER analysis, and their NMDS scores were calculated (Table 2). Rivers were primarily associated with Coleoptera taxa, whereas springs were associated with several groupings, including arachnids, gastropods, and isopods (Figure 2).

**Table 2:** Key invertebrate groups, SIMPER and NMDS analysis

	SIMPER % contribution to the habitat type effect	NMDS1 score	NMDS2 score	Functional feeding group
Isopoda, Amphisopidae *Phreatomerus latipes*	0.0532 (*SD* 0.0125)	0.6512	−0.0601	Shredders, gathering collectors
Gastropoda, Hydrobiidae *Fonscochlea* spp	0.0532 (*SD* 0.0125)	0.6512	−0.0601	Scrapers
Gastropoda, Hydrobiidae *Trochidrobia* spp	0.0532 (*SD* 0.0125)	0.6512	−0.0601	Scrapers
Diptera, Ceratopogonidae	0.0532 (*SD* 0.0125)	0.6512	−0.0601	Predators, gathering collectors, scrapers
Diptera, Chironomidae *Chironominae*	0.0532 (*SD* 0.0125)	0.6512	−0.0601	Gathering and filtering collectors, predators, shredders, scrapers
Coleoptera, Hydrophilidae *Berosus* spp (larv.)	0.0532 (*SD* 0.0125)	−0.5613	0.0056	Predators
Araneae, Lycosidae (Arachnida)	0.0390 (*SD* 0.0282)	0.4438	0.1279	Predators
Coleoptera, Dytiscidae *Eretes australis*	0.0345 (*SD* 0.0268)	−0.5827	−0.0513	Predators
Coleoptera, Dytiscidae *Necterosoma* spp	0.0334 (*SD* 0.0299)	−0.2694	−0.0216	Predators
Coleoptera, Dytiscidae *Limbodessus* spp	0.0312 (*SD*. 0.0274)	−0.3265	0.2921	Predators
Coleoptera, Hydrophilidae *Unknown* spp	0.0290 (*SD* 0.0290)	−0.1632	−0.1964	Shredder

**Figure 3:**
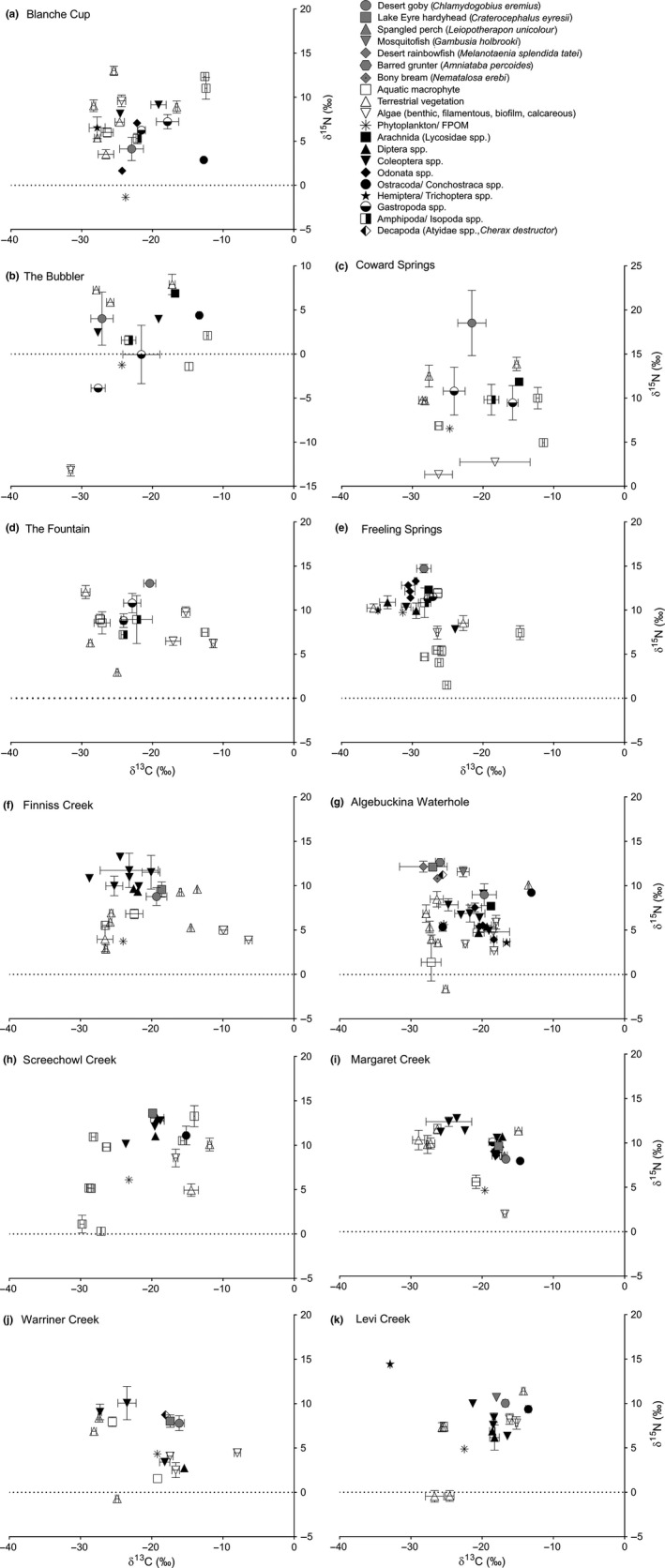
Stable isotope plots across sites (a–k), as of April 2014. Error bars represent one standard deviation in the mean score for that taxa. Error bars were omitted where insufficient individuals/biomass was available for three independent measures

**Figure 4:**
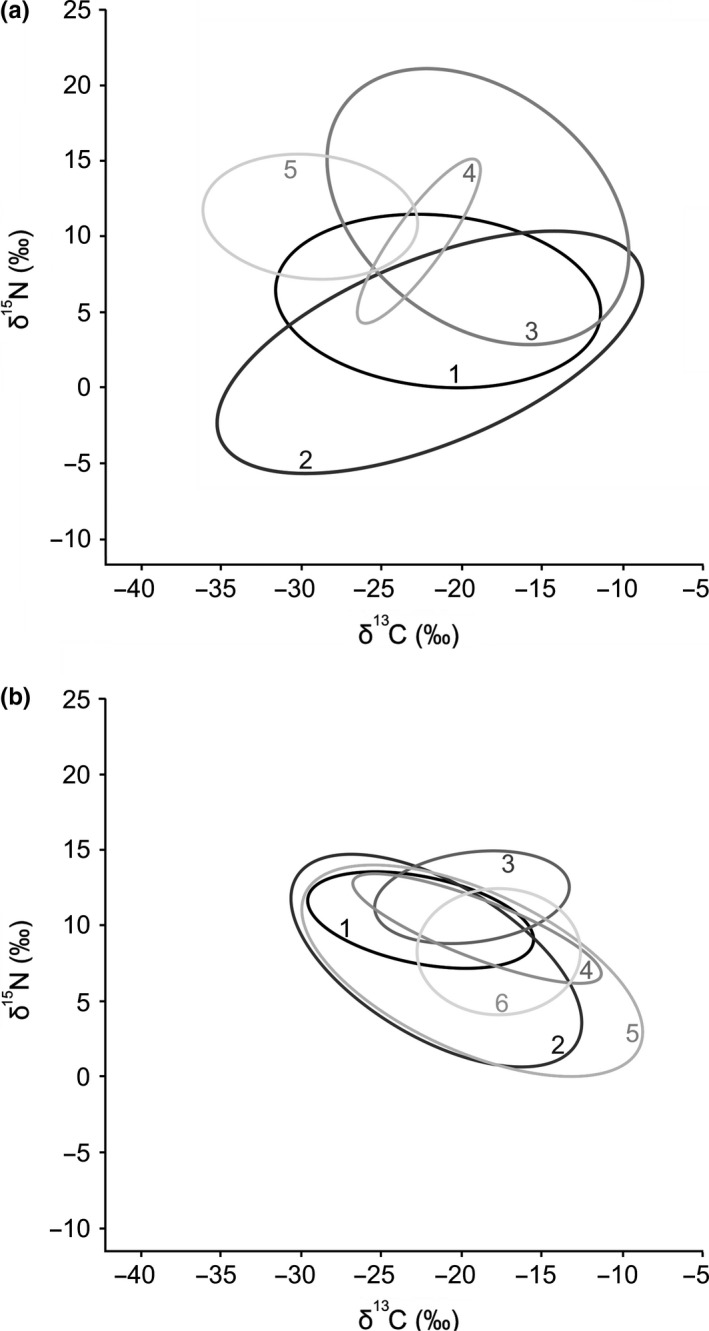
Standard ellipse areas based on 95% prediction intervals across (a) spring habitats and (b) river habitats. Spring site ellipses are differentiated according to shade and number, in order from 1. Blanche Cup (dark black), 2. The Bubbler, 3. Coward Springs, 4. The Fountain, to 5. Freeling Springs (light gray). River site ellipses are similarly differentiated according to shade, in order from 1. Finniss Creek (dark black), 2. Algebuckina Waterhole, 3. Screechowl Creek, 4. Margaret Creek, 5. Warriner Creek, to 6. Levi Creek (light gray)

Isotope plots across spring sites (Figure 3a–e) show apparent differences in basal resources, such as filamentous algae at The Bubbler (δ^15^N = −13.20‰ [*SD* 0.64], δ^13^C = −31.54‰ [*SD* 0.31]) and phytoplankton at Blanche Cup (δ^15^N = −1.35‰, δ^13^C = −23.79‰). Standard ellipse plots (Figure 4a) and the high among‐site variation in δ^15^N and δ^13^C values relative to river sites (Table 3) indicate variability in carbon sources and isotopic niches between springs.

**Table 3:** Analysis of animal food‐web characteristics by site, April 2014

Site	Total number of taxa	Corrected ellipse area (SEAc)			δ15N Adjusted mean and range (‰)	δ13C Mean and range (‰)
		Nonpredatory invertebrates	Predatory invertebrates and fish	All animals		
Blanche Cup	13	28.9196	29.5561	34.2473	−0.6319 (range = 7.4871)	−21.5157 (range = 15.0809)
The Bubbler	12	58.8392	42.5405	52.5168	4.1906 (range = 10.7520)	−22.0875 (range = 14.3445)
Coward Springs	6	2.0853	a	56.3905	5.2625 (range = 9.0569)	−19.0385 (range = 9.1887)
The Fountain	9	5.3309	a	7.1458	2.1159 (range = 5.8225)	−22.7351 (range = 3.7478)
Freeling Springs	15	4.9830	22.7581	15.3107	3.2957 (range = 6.8801)	−29.4565 (range = 10.9020)
Finniss Creek	12	1.2662	13.3044	12.0191	5.3571 (range = 4.4746)	−22.6357 (range = 10.1431)
Algebuckina	20	55.7328	18.2851	27.9873	3.7092 (range = 9.0442)	−21.6240 (range = 15.1613)
Screechowl Creek	7	6.2202	1.2875	11.4238	4.6727 (range = 3.4637)	−19.4025 (range = 8.4726)
Margaret Creek	15	15.3502	7.6874	9.0739	4.1933 (range = 4.8095)	−19.1181 (range = 11.1474)
Warriner Creek	10	59.1374	3.2488	38.096	2.7114 (range = 7.3205)	−19.4187 (range = 11.8375)
Levi Creek^b^	7	a	9.3009	12.8956	2.3955 (range = 4.5242)	−17.7366 (range = 7.7868)

a Insufficient data points to calculate SEAc.

b A single Hemiptera individual was excluded, as isotope values suggest it is a recent immigrant and unrelated to local food‐web conditions.

Isotope plots of river sites (Figure 3f–k) and mean δ^13^C values (Table 3) show animal communities clustered around −20‰, and standard ellipse plots (Figure 4b) showed that there was a high degree of isotopic niche overlap centered in around this value. This appears to be associated with riverine algae (mostly filamentous algae and benthic mats), which commonly had δ^13^C values from −15 to −20‰ (median = −16.38‰, mean = −14.99 [*SD* 4.46]). At several river sites, δ^13^C was below −25‰, likely reflecting inputs of terrestrial vegetation, which commonly had δ^13^C values from −25 to −30‰ (median = −26.35‰; mean = −24.01‰ [*SD* 5.40]).

For the animal communities at springs and river sites, there were no overall differences in SEAc, δ^15^N mean/range, δ^13^C mean/range, or total taxa richness based on habitat type (Table 3). Among spring sites, the largest SEAc and δ^15^N ranges were recorded at The Bubbler, suggesting high diet generalization and longer food chains, followed by Blanche Cup and Coward Springs. Among river sites, the largest and most persistent site, Algebuckina Waterhole, had the highest range of δ^15^N (indicating longer food chains) and the largest number of taxa, whereas the more ephemeral Warriner Creek exhibited the largest SEAc niches (indicating higher diet generalization).

The SEAc of functional groupings (Table 3) showed that the isotopic niche space occupied by predatory invertebrates and fish was larger in springs than river sites (*F*_1,7_ = 18.09, *p* = .0038), despite spring sites tending to have fewer predatory invertebrate taxa and fish. Although this should be interpreted cautiously as two spring sites did not have sufficient taxa to calculate SEAc for this group. There was no significant habitat difference for nonpredatory invertebrates.

## 4 DISCUSSION

Invertebrate assemblages across a wide geographical range in the western LEB were strongly associated with their local habitat type. Springs were linked with taxa that have low active dispersal capability and are less likely to be adept at coping with physically variable environments (i.e., isopods and gastropods), whereas rivers were linked to Coleoptera taxa with the capacity for active flight and dispersal (Boda & Csabai, 2013). Temporal patterns in hydrology and connectivity have been shown to be an important driver of invertebrate assemblages and diversity in the arid Cooper Creek (Marshall, Sheldon, Thoms, & Choy, 2006), so the possible flow event within 2 months of sampling may have been a factor driving the observed similarity between river waterholes. Directly following a flow disturbance and the re‐wetting of dry or drying waterholes, species succession can favor dispersive invertebrates, and in this case, Coleopterans may represent early colonizers (Sousa, 1979; Ward, Tockner, Arscott, & Claret, 2002).

While dispersal strategies appear to allow certain taxa to persist across more physically variable LEB rivers, in temperate streams and isolated pools on the Iberian Peninsula, the opposite pattern was observed, with lentic waterbodies linked to dispersive Coleopteran species (Ribera & Vogler, 2000). Nonetheless, the mechanism underlying this is consistent with what we observed, as Ribera and Vogler's isolated pools were also more ephemeral and required frequent migration for aquatic species, so may be considered more like LEB waterholes. Consistent findings from the Pilbara region in northwest Australia show that the local physical conditions and connectivity drive invertebrate assemblages in arid waterholes (Moran‐Ordonez et al., 2015). These results reinforce the ecological importance of the broad interconnected network of ephemeral river channels to the persistence of certain aquatic taxa in arid landscapes (Mossop et al., 2015).

Our results support previous studies showing permanent groundwater is vital to the persistence and evolution of certain endemic aquatic taxa in this arid landscape (Brim‐Box et al., 2014, 2008; Davis et al., 2013; Murphy, Guzik, Cooper, & Austin, 2015), including isopods and gastropods that show catchment‐ or spring‐specific endemism (Guzik, Adams, Murphy, Cooper, & Austin, 2012; Murphy et al., 2012). This is also true of terrestrial wolf spiders (Araneae, Lycosidae), which are dominant invertebrate predators in GAB springs and were commonly detected in aquatic invertebrate samples (Gotch, Adams, Murphy, & Austin, 2008).

Patterns in fish distributions similarly support the dual ecological significance of permanent and ephemeral habitats, where mound springs are key refuges for the poor swimming desert goby, which shows genetic structuring separated according to the north and south catchments of Lake Eyre (Mossop et al., 2015). Conversely, more actively dispersive species such as spangled perch (*Leiopotherapon unicolor*) and the Lake Eyre hardyhead (*Craterocephalus eyresii*) are widespread in hydrologically variable riverine habitats across the LEB without reliance on the spring habitats (McNeil et al., 2011).

Food webs showed distinct similarity across river habitats, with the isotopic niche of animal communities overlapping and appearing clustered around −20‰ δ^13^C. This suggests autochthonous algal food sources (particularly the algal benthic mat) may be important primary producers in rivers during no flow periods, although our sites were also affected by a relatively recent flow event, which can enrich the δ^13^C of benthic algae (Hadwen, Spears, & Kennard, 2010). Our results are consistent with those from the eastern Lake Eyre Basin where benthic algal carbon was highly productive and a key carbon input during no‐flow periods in Cooper Creek (Bunn et al., 2003).

For Finniss Creek, Margaret Creek, Warriner Creek, and Algebuckina, the δ^13^C range of fauna was relatively large, ranging to above −25‰. This suggests that terrestrial vegetation may also be providing significant inputs, and it is expected that both terrestrial and autochthonous sources are providing carbon inputs into rivers (Robertson et al., 1999). The apparent importance of autochthonous carbon to river sites is consistent with international models (see Thorp & Delong, 1994) and studies of Australian rivers under prolonged low‐flow/ no‐flow periods (Bunn et al., 2003; Gawne et al., 2007). The extreme hydrological variability of LEB rivers likely facilitates floodplain connection and terrestrial inputs, but for the majority of time, these rivers exist as nonflowing isolated pools and no‐flow droughts can persist for multiple years (Kotwicki & Allan, 1998). Our findings suggest that consistent autochthonous algal productivity is an important for aquatic communities to persist despite the unpredictable flow regime.

Across springs, the basal carbon sources supporting food webs appear to vary based on their local environment. An unusual example is the filamentous algae in The Bubbler, which had an extremely low δ^15^N value. This has been observed elsewhere, where phosphorus limitation in lichens and mangroves encourages the uptake of isotopically depleted atmospheric ammonia (Fogel et al., 2008), suggesting nutrient limitation and atmospheric nitrogen fixing are potentially influencing basal productivity at this spring. This is also potentially influencing productivity by phytoplankton/FPOM sources at Blanche Cup, which is likely a significant source of basal carbon to this food web. Gastropods appear particularly important to spring food webs, as they are hugely abundant (usually >1,000 per sample) and our own qualitative observations of desert goby gut contents (the only vertebrate species occupying spring sites) suggest that gastropods make up a significant portion of their diet. An important role of gastropods demonstrates that inputs from biofilm and algae are key basal sources underpinning these food webs, which further emphasizes the value of stable isotopes in exploring patterns of primary productivity, particularly in aquatic ecosystems, and importance of basal resources to aquatic community structure.

Contrary to our hypotheses, food webs from contrasting habitat types did not appear to have overall differences in their structural attributes. Neither the SEAc nor δ^15^N range was larger across the physically stable springs, suggesting that the buffer against extreme hydrological variability provided by groundwater does not necessarily influence these general food‐web measures. While the disturbance hypothesis predicts that longer food chains are more sensitive to perturbations (McHugh et al., 2010), our data are more consistent with analysis showing that disturbance does not appear to have an overarching effect of food‐chain length (Takimoto & Post, 2013). This is analogous with theoretical food‐web modeling, which has suggested that larger complex food webs will be more sensitive to perturbations (May, 1972). More recent modeling instead suggests that larger complex food webs are not inherently unstable, by incorporating characteristics of empirical communities such as body size scaling (Brose, Williams, & Martinez, 2006) and adaptive behavior (Valdovinos, Ramos‐Jiliberto, Garay‐Narvaez, Urbani, & Dunne, 2010). Finally, the two largest δ^15^N ranges were detected at the largest spring and river sites, suggesting that the area/volume of the aquatic habitat is likely to play a role in the food‐chain length. The size and spatial heterogeneity of stream ecosystems has been shown to influence food‐web structures such as food‐chain length (Spencer & Warren, 1996; Thompson & Townsend, 2005; consistent with hypotheses such as the Productive Space Hypothesis, Schoener, 1989), and among‐site variation in physical structure may significantly influence the variability in spring and river food webs.

## CONFLICT OF INTEREST

We declare no conflict of interests in relation to this manuscript.

## AUTHOR CONTRIBUTIONS

NPM wrote the main manuscript and prepared all figures and tables. All authors contributed to the formulation and execution of the experiments described therein, and all authors reviewed the manuscript.

## Data Availability

The data that support the findings of this study are available from the Dryad Digital Repository (Moran, Wong, & Thompson, 2019, https://doi.org/10.5061/dryad.j18jt4n).

## References

[ece35648-bib-0001] Binzer, A. , Guill, C. , Brose, U. , & Rall, B. C. (2012). The dynamics of food chains under climate change and nutrient enrichment. Philosophical Transactions of the Royal Society B: Biological Sciences, 367, 2935–2944. 10.1098/rstb.2012.0230 PMC3479739 23007081

[ece35648-bib-0002] Boda, P. , & Csabai, Z. (2013). When do beetles and bugs fly? A unified scheme for describing seasonal flight behaviour of highly dispersing primary aquatic insects. Hydrobiologia, 703, 133–147. 10.1007/s10750-012-1350-3

[ece35648-bib-0003] Brim‐Box, J. , Davis, J. , Strehlow, K. , McBurnie, G. , Duguid, A. , Brock, C. , … Palmer, C. (2014). Persistence of central Australian aquatic invertebrate communities. Marine and Freshwater Research, 65, 562–572. 10.1071/MF13131

[ece35648-bib-0004] Brim‐Box, J. , Duguid, A. , Read, R. E. , Kimber, R. G. , Knapton, A. , Davis, J. , & Bowland, A. E. (2008). Central Australian waterbodies: The importance of permanence in a desert landscape. Journal of Arid Environments, 72, 1395–1413. 10.1016/j.jaridenv.2008.02.022

[ece35648-bib-0005] Brose, U. , Williams, R. J. , & Martinez, N. D. (2006). Allometric scaling enhances stability in complex food webs. Ecology Letters, 9, 1228–1236. 10.1111/j.1461-0248.2006.00978.x 17040325

[ece35648-bib-0006] Brown, V. K. , & Southwood, T. R. E. (1983). Trophic diversity, niche breadth and generation times of exopterygote insects in a secondary succession. Oecologia, 56, 220–225. 10.1007/BF00379693 28310197

[ece35648-bib-0007] Bunn, S. E. , & Arthington, A. H. (2002). Basic principles and ecological consequences of altered flow regimes for aquatic biodiversity. Environmental Management, 30, 492–507. 10.1007/s00267-002-2737-0 12481916

[ece35648-bib-0008] Bunn, S. E. , Davies, P. M. , & Winning, M. (2003). Sources of organic carbon supporting the food web of an arid zone floodplain river. Freshwater Biology, 48, 619–635. 10.1046/j.1365-2427.2003.01031.x

[ece35648-bib-0009] Cole, J. J. , Carpenter, S. R. , Pace, M. L. , Van de Bogert, M. C. , Kitchell, J. L. , & Hodgson, J. R. (2006). Differential support of lake food webs by three types of terrestrial organic carbon. Ecology Letters, 9, 558–568. 10.1111/j.1461-0248.2006.00898.x 16643301

[ece35648-bib-0010] Connell, J. H. (1978). Diversity in tropical rain forests and coral reefs – High diversity of trees and corals is maintained only in a non‐equilibrium state. Science, 199, 1302–1310. 10.1126/science.199.4335.1302 17840770

[ece35648-bib-0011] Costelloe, J. F. , Grayson, R. B. , McMahon, T. A. , & Argent, R. M. (2005). Spatial and temporal variability of water salinity in an ephemeral, arid‐zone river, central Australia. Hydrological Processes, 19, 3147–3166. 10.1002/hyp.5837

[ece35648-bib-0012] Costelloe, J. F. , & Russell, K. L. (2014). Identifying conservation ties for aquatic refugia in an arid zone, ephemeral catchment: A hydrological approach. Ecohydrology, 7, 1534–1544. 10.1002/eco.1476

[ece35648-bib-0013] Davis, J. , Pavlova, A. , Thompson, R. , & Sunnucks, P. (2013). Evolutionary refugia and ecological refuges: Key concepts for conserving Australian arid zone freshwater biodiversity under climate change. Global Change Biology, 19, 1970–1984. 10.1111/gcb.12203 23526791 PMC3746109

[ece35648-bib-0014] Davis, J. , Sim, L. , Thompson, R. M. , Pinder, A. , Box, J. B. , Murphy, N. P. , … Sunnucks, P. (2018). Patterns and drivers of aquatic invertebrate diversity across an arid biome. Ecography, 41, 375–387. 10.1111/ecog.02334

[ece35648-bib-0015] Dunne, J. A. , Saleska, S. R. , Fischer, M. L. , & Harte, J. (2004). Integrating experimental and gradient methods in ecological climate change research. Ecology, 85, 904–916. 10.1890/03-8003

[ece35648-bib-0016] Fogel, M. L. , Wooller, M. J. , Cheeseman, J. , Smallwood, B. J. , Roberts, Q. , Romero, I. , & Meyers, M. J. (2008). Unusually negative nitrogen isotopic compositions (δ^15^N) of mangroves and lichens in an oligotrophic, microbially‐influenced ecosystem. Biogeosciences, 5, 1693–1704.

[ece35648-bib-0017] Fox, J. W. (2013). The intermediate disturbance hypothesis should be abandoned. Trends in Ecology and Evolution, 28, 86–92. 10.1016/j.tree.2012.08.014 22981468

[ece35648-bib-0018] Gawne, B. , Merrick, C. , Williams, D. G. , Rees, G. , Oliver, R. , Bowen, P. M. , … Lorenz, Z. (2007). Patterns of primary and heterotrophic productivity in an arid lowland river. River Research and Applications, 23, 1070–1087. 10.1002/rra.1033

[ece35648-bib-0019] Gotch, T. B. , Adams, M. , Murphy, N. P. , & Austin, A. D. (2008). A molecular systematic overview of wolf spiders associated with Great Artesian Basin springs in South Australia: Evolutionary affinities and an assessment of metapopulation structure in two species. Invertebrate Systematics, 22, 151–165. 10.1071/IS07045

[ece35648-bib-0020] Guzik, M. T. , Adams, M. A. , Murphy, N. P. , Cooper, S. J. B. , & Austin, A. D. (2012). Desert springs: Deep phylogeographic structure in an ancient endemic Crustacean (*Phreatomerus latipes*). PLoS One, 7, e37642 10.1371/journal.pone.0037642 22815684 PMC3398905

[ece35648-bib-0021] Hadwen, W. L. , Fellows, C. S. , Westhorpe, D. P. , Rees, G. N. , Mitrovic, S. M. , Taylor, B. , … Croome, R. (2010). Longitudinal trends in river functioning: Patterns of nutrient and carbon processing in three Australian rivers. River Research and Applications, 26, 1129–1152. 10.1002/rra.1321

[ece35648-bib-0022] Hadwen, W. L. , Spears, M. , & Kennard, M. J. (2010). Temporal variability of benthic algal δ^13^C signatures influences assessments of carbon flows in stream food webs. Hydrobiologia, 651, 239–251. 10.1007/s10750-010-0303-y

[ece35648-bib-0023] Hawking, J. H. , Smith, L. , LeBusque, K. , & Davey, C. (Eds.) (2013). Identification and ecology of Australian freshwater invertebrates. Retrieved from http://www.mdfrc.org.au/bugguide

[ece35648-bib-0024] Ho, S. S. , Bond, N. R. , & Lake, P. S. (2011). Comparing food‐web impacts of a native invertebrate and an invasive fish as predators in small floodplain wetlands. Marine and Freshwater Research, 62, 372–382. 10.1071/MF10222

[ece35648-bib-0025] Jackson, A. L. , Inger, R. , Parnell, A. C. , & Bearhop, S. (2011). Comparing isotopic niche widths among and within communities: SIBER – Stable Isotope Bayesian Ellipses in R. Journal of Animal Ecology, 80, 595–602. 10.1111/j.1365-2656.2011.01806.x 21401589

[ece35648-bib-0026] Kondoh, M. (2003). Foraging adaptation and the relationship between food‐web complexity and stability. Science, 299, 1388–1391. 10.1126/science.1079154 12610303

[ece35648-bib-0027] Kotwicki, V. , & Allan, R. (1998). La Nina de Australia ‐ Contemporary and palaeo‐hydrology of Lake Eyre. Palaeogeography, Palaeoclimatology, Palaeoecology, 144, 265–280.

[ece35648-bib-0028] Krause, A. E. , Frank, K. A. , Mason, D. M. , Ulanowicz, R. E. , & Taylor, W. W. (2003). Compartments revealed in food‐web structure. Nature, 426, 282–285. 10.1038/nature02115 14628050

[ece35648-bib-0029] Kullberg, R. G. , & Scheibe, J. S. (1989). The effects of succession on niche breadth and overlap in a hot‐spring algal community. The American Midland Naturalist Journal, 121, 21–31. 10.2307/2425653

[ece35648-bib-0030] Lake, P. S. (2003). Ecological effects of perturbation by drought in flowing waters. Freshwater Biology, 48, 1161–1172. 10.1046/j.1365-2427.2003.01086.x

[ece35648-bib-0031] Layer, K. , Hildrew, A. G. , Jenkins, G. B. , Riede, J. O. , Rossiter, S. J. , Townsend, C. R. , & Woodward, G. (2011). Long‐term dynamics of a well‐characterised food web: Four decades of acidification and recovery in the Broadstone Stream model System In Woodward G. (Ed.), Advances in ecological research (Vol. 44, pp. 69‐117). Amsterdam, The Netherlands: Academic Press.

[ece35648-bib-0032] Layman, C. A. , Araujo, M. S. , Boucek, R. , Hammerschlag‐Peyer, C. M. , Harrison, E. , Jud, Z. R. , … Bearhop, S. (2012). Applying stable isotopes to examine food‐web structure: An overview of analytical tools. Biological Reviews, 87, 545–562. 10.1111/j.1469-185X.2011.00208.x 22051097

[ece35648-bib-0033] Ledger, M. E. , Brown, L. E. , Edwards, F. K. , Milner, A. M. , & Woodward, G. (2013). Drought alters the structure and functioning of complex food webs. Nature Climate Change, 3, 223–227. 10.1038/nclimate1684

[ece35648-bib-0034] Marshall, J. C. , Sheldon, F. , Thoms, M. , & Choy, S. (2006). The macroinvertebrate fauna of an Australian dryland river: Spatial and temporal patterns and environmental relationships. Marine and Freshwater Research, 57, 61–74. 10.1071/MF05021

[ece35648-bib-0035] May, R. M. (1972). Will a large complex system be stable. Nature, 238, 413–414. 10.1038/238413a0 4559589

[ece35648-bib-0036] McGill, B. J. , Enquist, B. J. , Weiher, E. , & Westoby, M. (2006). Rebuilding community ecology from functional traits. Trends in Ecology and Evolution, 21, 178–185. 10.1016/j.tree.2006.02.002 16701083

[ece35648-bib-0037] McHugh, P. A. , McIntosh, A. R. , & Jellyman, P. G. (2010). Dual influences of ecosystem size and disturbance on food chain length in streams. Ecology Letters, 13, 881–890. 10.1111/j.1461-0248.2010.01484.x 20482579

[ece35648-bib-0038] McNeil, D. G. , Schmarr, D. W. , & Rosenberger, A. E. (2011). Climatic variability, fish and the role of refuge waterholes in the Neales River Catchment: Lake Eyre Basin, South Australia. South Australian Research and Development Institute (Aquatic Sciences) report to the South Australian Arid Lands NRM Board, Port Augusta.

[ece35648-bib-0039] Menge, B. A. , & Sutherland, J. P. (1987). Community regulation – Variation in disturbance, competition, and predation in relation to environmental‐stress and recruitment. The American Naturalist, 130, 730–757. 10.1086/284741

[ece35648-bib-0040] Moran, N. P. , Wong, B. B. M. , & Thompson, R. M. (2019). Data from: Communities at the extreme: Aquatic food webs in desert landscapes Dryad Digital Repository, 10.5061/dryad.j18jt4n PMC6802011 31641486

[ece35648-bib-0041] Moran‐Ordonez, A. , Pavlova, A. , Pinder, A. M. , Sim, L. , Sunnucks, P. , Thompson, R. M. , & Davis, J. (2015). Aquatic communities in arid landscapes: Local conditions, dispersal traits and landscape configuration determine local biodiversity. Diversity and Distributions, 21, 1230–1241. 10.1111/ddi.12342

[ece35648-bib-0042] Mossop, K. D. , Adams, M. , Unmack, P. J. , Smith Date, K. L. , Wong, B. B. M. , & Chapple, D. G. (2015). Dispersal in the desert: Ephemeral water drives connectivity and phylogeography of an arid‐adapted fish. Journal of Biogeography, 42, 2374–2388. 10.1111/jbi.12596

[ece35648-bib-0043] Mudd, G. M. (2000). Mound springs of the Great Artesian Basin in South Australia: A case study from Olympic Dam. Environmental Geology, 39, 463–476. 10.1007/s002540050452

[ece35648-bib-0045] Murphy, N. P. , Adams, M. , & Austin, A. D. (2009). Independent colonization and extensive cryptic speciation of freshwater amphipods in the isolated groundwater springs of Australia's Great Artesian Basin. Molecular Ecology, 18, 109–122. 19140968 10.1111/j.1365-294X.2008.04007.x

[ece35648-bib-0046] Murphy, N. P. , Breed, M. F. , Guzik, M. T. , Cooper, S. J. B. , & Austin, A. D. (2012). Trapped in desert springs: Phylogeography of Australian desert spring snails. Journal of Biogeography, 39, 1573–1582. 10.1111/j.1365-2699.2012.02725.x

[ece35648-bib-0047] Murphy, N. P. , Guzik, M. T. , Cooper, S. J. B. , & Austin, A. D. (2015). Desert spring refugia: Museums of diversity or evolutionary cradles? Zoologica Scripta, 44, 693–701. 10.1111/zsc.12129

[ece35648-bib-0048] Newsome, S. D. , Martinez del Rio, C. , Bearhop, S. , & Phillips, D. L. (2007). A niche for isotopic ecology. Frontiers in Ecology and the Environment, 5, 429–436. 10.1890/1540-9295(2007)5[429:ANFIE]2.0.CO;2

[ece35648-bib-0049] Oksanen, J. , Kindt, R. , Legendre, P. , O'Hara, B. , Stevens, M. H. H. , Oksanen, M. J. , & Suggests, M. (2007). The vegan package. Community Ecology Package, 10, 631–637.

[ece35648-bib-0050] Pimm, S. L. , & Lawton, J. H. (1977). Number of trophic levels in ecological communities. Nature, 268, 329–331. 10.1038/268329a0

[ece35648-bib-0051] Post, D. M. (2002). Using stable isotopes to estimate trophic position: Models, methods, and assumptions. Ecology, 83, 703–718. 10.1890/0012-9658(2002)083[0703:USITET]2.0.CO;2

[ece35648-bib-0052] R Core Team (2018). R: A language and environment for statistical computing. Vienna, Austria: R Foundation for Statistical Computing.

[ece35648-bib-0053] Ribera, I. , & Vogler, A. P. (2000). Habitat type as a determinant of species range sizes: The example of lotic–lentic differences in aquatic Coleoptera. Biological Journal of the Linnean Society, 71, 33–52.

[ece35648-bib-0054] Robertson, A. I. , Bunn, S. E. , Boon, P. I. , & Walker, K. F. (1999). Sources, sinks and transformations of organic carbon in Australian floodplain rivers. Marine and Freshwater Research, 50, 813–829. 10.1071/MF99112

[ece35648-bib-0055] Sabo, J. L. , Finlay, J. C. , Kennedy, T. , & Post, D. M. (2010). The role of discharge variation in scaling of drainage area and food chain length in rivers. Science, 330, 965–967. 10.1126/science.1196005 20947729

[ece35648-bib-0056] Schoener, T. W. (1989). Food webs from the small to the large. Ecology, 70, 1559–1589.

[ece35648-bib-0057] Sousa, W. P. (1979). Experimental investigations of disturbance and ecological succession in a rocky inter‐tidal algal community. Ecological Monographs, 49, 227–254. 10.2307/1942484

[ece35648-bib-0058] Spencer, M. , & Warren, P. H. (1996). The effects of habitat size and productivity on food web structure in small aquatic microcosms. Oikos, 75, 419–430. 10.2307/3545882

[ece35648-bib-0059] Takimoto, G. , & Post, D. M. (2013). Environmental determinants of food‐chain length: A meta‐analysis. Ecological Research, 28, 675–681. 10.1007/s11284-012-0943-7

[ece35648-bib-0060] Thompson, R. M. , Brose, U. , Dunne, J. A. , Hall, R. O. , Hladyz, S. , Kitching, R. L. , … Tylianakis, J. M. (2012). Food webs: Reconciling the structure and function of biodiversity. Trends in Ecology and Evolution, 27, 689–697. 10.1016/j.tree.2012.08.005 22959162

[ece35648-bib-0061] Thompson, R. M. , Dunne, J. A. , & Woodward, G. (2012). Freshwater food webs: Towards a more fundamental understanding of biodiversity and community dynamics. Freshwater Biology, 57, 1329–1341. 10.1111/j.1365-2427.2012.02808.x

[ece35648-bib-0062] Thompson, R. M. , & Townsend, C. R. (2005). Energy availability, spatial heterogeneity and ecosystem size predict food‐web structure in streams. Oikos, 108, 137–148. 10.1111/j.0030-1299.2005.11600.x

[ece35648-bib-0063] Thorp, J. H. , & Delong, M. D. (1994). The riverine productivity model ‐ An heuristic view of carbon‐sources and organic‐processing in large river ecosystems. Oikos, 70, 305–308. 10.2307/3545642

[ece35648-bib-0064] Townsend, C. R. , Thompson, R. M. , McIntosh, A. R. , Kilroy, C. , Edwards, E. , & Scarsbrook, M. R. (1998). Disturbance, resource supply, and food‐web architecture in streams. Ecology Letters, 1, 200–209. 10.1046/j.1461-0248.1998.00039.x

[ece35648-bib-0065] Valdovinos, F. S. , Ramos‐Jiliberto, R. , Garay‐Narvaez, L. , Urbani, P. , & Dunne, J. A. (2010). Consequences of adaptive behaviour for the structure and dynamics of food webs. Ecology Letters, 13, 1546–1559. 10.1111/j.1461-0248.2010.01535.x 20937057

[ece35648-bib-0066] Vander Zanden, M. J. , Cabana, G. , & Rasmussen, J. B. (1997). Comparing trophic position of freshwater fish calculated using stable nitrogen isotope ratios (δ^15^N) and literature dietary data. Canadian Journal of Fisheries and Aquatic Sciences, 54, 1142–1158.

[ece35648-bib-0067] Walters, A. W. , & Post, D. M. (2008). An experimental disturbance alters fish size structure but not food chain length in streams. Ecology, 89, 3261–3267. 10.1890/08-0273.1 19137932

[ece35648-bib-0068] Ward, J. V. , Tockner, K. , Arscott, D. B. , & Claret, C. (2002). Riverine landscape diversity. Freshwater Biology, 47, 517–539. 10.1046/j.1365-2427.2002.00893.x

[ece35648-bib-0069] Wootton, J. T. (1994a). The nature and consequences of indirect effects in ecological communities. Annual Review of Ecology and Systematics, 25, 443–466. 10.1146/annurev.ecolsys.25.1.443

[ece35648-bib-0070] Wootton, J. T. (1994b). Predicting direct and indirect effects – An integrated approach using experiments and path‐analysis. Ecology, 75, 151–165. 10.2307/1939391

[ece35648-bib-0071] Wootton, J. T. , Parker, M. S. , & Power, M. E. (1996). Effects of disturbance on river food webs. Science, 273, 1558–1561. 10.1126/science.273.5281.1558

