# Supplementary material for: Communities at the extreme: Aquatic food webs in desert landscapes
Source: Ecol Evol. 2019 Sep 12;9(19):11464–75. doi: 10.1002/ece3.5648 (PMC6802011; doi:10.1002/ece3.5648)

**Appendix 1**

Recorded rainfall at Bureau of Meteorology rainfall stations in the western Lake Eyre region is shown in Figure A1-A3. Stations were selected as the closest stations to study sites with data available in 2013-14 (see Fig. 1), with Oodnadatta Airport north of all study sites, Nilpinna between the northern Neales Catchment sites and north of the southern catchments’ sites, and Marree Aero south of all study sites. The significant rainfall peak in Feb 2014 appear to be more concentrated in southern areas, although all catchments included in the study would have received some significant rain during this period and the potential for surface water flow. (Data extraction details: A1- Bureau of Meteorology station number: 17043, Station name: OODNADATTA AIRPORT, Latitude (decimal degrees, south negative): -27.5, Longitude (decimal degrees, east positive): 135.45, Created on Fri 22 Feb 2019 08:36:44 AM GMT-00:00 from Climate Data Online, Bureau of Meteorology. http://www.bom.gov.au/climate/data; A2- Bureau of Meteorology station number: 17127, Station name: NILPINNA, Latitude (decimal degrees, south negative): -28.48, Longitude (decimal degrees, east positive): 135.92, Created on Fri 22 Feb 2019 08:36:50 AM GMT-00:00 from Climate Data Online, Bureau of Meteorology, http://www.bom.gov.au/climate/data; A3- Station name: MARREE AERO. Bureau of Meteorology station number: 17126, Latitude (decimal degrees, south negative): -29.66, Longitude (decimal degrees, east positive): 138.07, Created on Fri 22 Feb 2019 08:36:35 AM GMT-00:00 from Climate Data Online, Bureau of Meteorology. http://www.bom.gov.au/climate/data)

Figure A: Regional daily rainfall records 2013-14


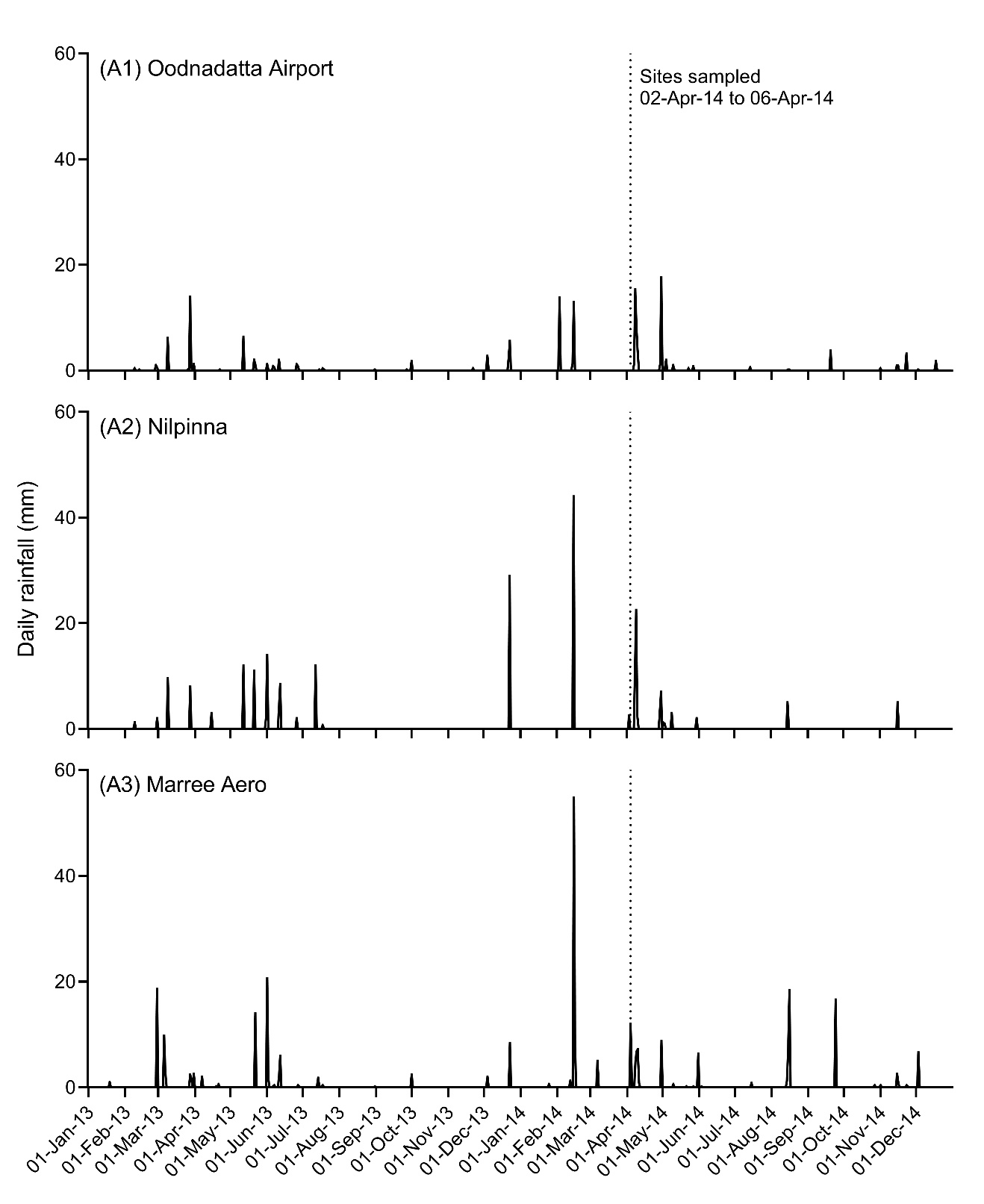

Supplement: Supplementary file 1 [file ECE3-9-11464-s001.docx]
